# Xist localization and function: new insights from multiple levels

**DOI:** 10.1186/s13059-015-0733-y

**Published:** 2015-08-15

**Authors:** Andrea Cerase, Greta Pintacuda, Anna Tattermusch, Philip Avner

**Affiliations:** EMBL Mouse Biology Unit, Monterotondo, 00015 (RM) Italy; Department of Biochemistry, University of Oxford, Oxford, OX1 3QU UK; Institut Pasteur, Unite de Genetique Moleculaire Murine, CNRS, URA2578 Paris, France

## Abstract

In female m ammals, one of the two X chromosomes in each cell is transcriptionally silenced in order to achieve dosage compensation between the genders in a process called X chromosome inactivation. The master regulator of this process is the long non-coding RNA Xist. During X-inactivation, Xist accumulates in cis on the future inactive X chromosome, triggering a cascade of events that provoke the stable silencing of the entire chromosome, with relatively few genes remaining active. How Xist spreads, what are its binding sites, how it recruits silencing factors and how it induces a specific topological and nuclear organization of the chromatin all remain largely unanswered questions. Recent studies have improved our understanding of Xist localization and the proteins with which it interacts, allowing a reappraisal of ideas about Xist function. We discuss recent advances in our knowledge of Xist-mediated silencing, focusing on Xist spreading, the nuclear organization of the inactive X chromosome, recruitment of the polycomb complex and the role of the nuclear matrix in the process of X chromosome inactivation.

## Introduction

X chromosome inactivation (XCI) is the mechanism that has evolved in eutherian mammals to ensure dosage compensation between *XX* (female) and *XY* (male) individuals. Dosage compensation depends on the efficient silencing of genes on one of the two X chromosomes in each cell of the female early in development. This process is crucially dependent on a specific locus on the X — the X inactivation center (XIC) — which includes, among other genetic elements, the *Xist* gene, which is necessary for the process of XCI [1]. *Xist* encodes a 17-kb long non-coding RNA (lncRNA) that, despite being capped, spliced and poly-adenylated, is retained in the nucleus.

In mouse, XCI occurs in two different fashions. During early embryogenesis, the paternal X is preferentially inactivated (imprinted XCI). At the blastula stage, in the cells of the inner cell mass, this imprinted XCI is reverted, and each chromosome in such cells has an equal chance to be inactivated (random XCI). Initiation of XCI is associated with the monoallelic upregulation of Xist and its spreading and coating in cis of the presumptive inactive X (initiation phase of XCI). This triggers a cascade of events, including the acquisition of repressive chromatin modifications, exclusion of RNA polymerase II (Pol II) and removal of active histone marks, histone exchange and DNA methylation. These events act in concert to ensure the stable repression of the entire chromosome and the maintenance of the silent state (maintenance phase of XCI) [2–5].

Although many studies have described various aspects of the underlying XCI mechanism, we are far from having a complete understanding of the process, especially at the molecular level. For example, we currently still do not have definitive answers to questions such as how Xist triggers silencing, how it recruits chromatin remodelers or how the silent state is maintained.

Here, we review recent progress in the field, pointing out the strengths, weaknesses and inconsistencies of recent findings. In particular, we highlight recent evidence indicating that chromosomal topology, nuclear organization, and chromatin accessibility all have key roles in the XCI process [6].

### Xist spreading and nuclear organization of the inactive X chromosome

Two recently published studies have shed light on Xist spreading and localization [7, 8] (and are commented upon elsewhere [9, 10]). Taking advantage of labeled probes complementary to Xist, pulldowns of Xist-associated chromatin at different stages of XCI were obtained and analyzed by next-generation DNA sequencing [capture hybridization analysis of RNA targets (CHART) and RNA antisense purification-sequencing (RAP-Seq); Box 1]. The studies cover both the initiation phase [recapitulated in differentiating female embryonic stem cells (ESCs) and male inducible-*Xist* ESCs], and the maintenance phase of XCI (studied in fully differentiated female fibroblasts; Box 1). Importantly, the different experimental systems used were complementary, compensating for the potential limitations of each system. For instance, in the male inducible-*Xist* cell lines used by Engreitz and colleagues [7], *Xist* upregulation can be both more rapid and intense than that occurring at the endogenous *Xist* locus. It is also possible that early time-points in the inducible systems correspond to relatively late time-points in differentiating female ESC lines [11, 12]. Finally, *Xist* upregulation in the inducible system is both well synchronized and relatively homogeneous [11], whereas ex vivo differentiation of ESC systems is often both asynchronous and non-homogeneous [6].

The principle result from both papers is the observation that Xist localization is initially restricted to a few discrete genomic locations, before extending more broadly over the entire chromosome. Xist coating starts within silent gene-dense regions and then proceeds to spread to active genes on the entire presumptive Xi. Both studies [7, 8] also confirmed that Xist accumulation at active genes requires the presence of Xist A-repeats, a class of structurally conserved repeats, previously identified as necessary for Xist-mediated silencing [12].

**Fig. 1:**
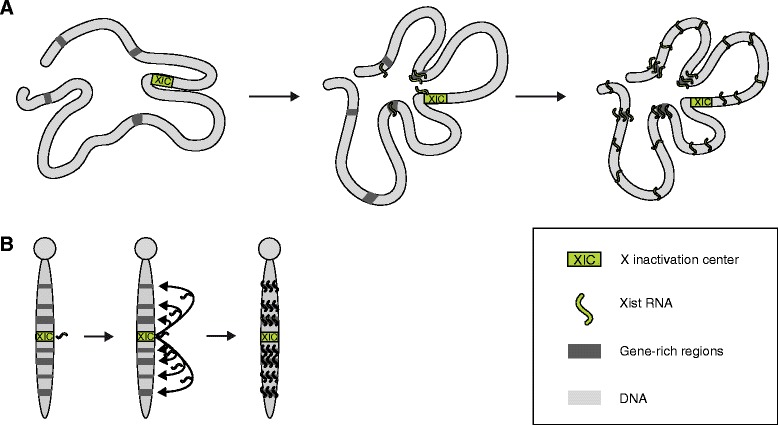
Models of the localization and spreading of Xist. **a** Three-dimensional spreading model of Xist localization. Xist might use close-proximity sites for its initial spreading (*left* and *middle panels*) before accumulating over the whole chromosome. At the final stages of spreading, Xist shows the highest enrichment at gene-rich regions (*right panel*). **b** Linear model of Xist spreading showing a classical representation of Xist decorating G-light bands on metaphase chromosomes

The two studies concur to show that, once spread, Xist is associated with gene-rich, open chromatin regions (high-affinity sites), which are enriched for the presence of short interspersed nuclear elements (SINEs) and anti-correlated with the presence of long interspersed nuclear elements (LINEs) and lamin interaction sites [7, 8]. These observations are in keeping with older cytological evidence suggesting a strong association of Xist localization with G-light bands (gene-rich regions), with gene-poor regions representing, predominantly, Xist low-affinity sites [13, 14] (Fig. 1a).

Strikingly, both papers highlight a strong dependence of Xist localization on the relative positioning of the *Xist* locus (XIC). Engreitz and colleagues [7] elegantly showed that moving the inducible *Xist* integration site from its endogenous location to that of the *Hprt* locus leads to a substantial change in the initial contact sites of Xist accumulation. The new contact sites correlate with the high-throughput chromosome conformation capture (Hi-C) interactions of the new locus [15–17] (Box 1). This strongly suggests that Xist exploits genomic proximity and topology in order to spread in cis, rather than depends on the presence of particular consensus sequences, as occurs in *Caenorhabditis elegans* [18] or in *Drosophila* [19]. Interestingly, the studies used XIC-centered HiC datasets obtained from a male cell line as a reference for identifying regions that are in close proximity, suggesting that the initial steps do not depend on female-specific identifiers [16, 17].

The observation that early localization sites depend on the location of *Xist* and appear to be exclusively position dependent implies that, at least initially, the first contact sites are not necessarily high-affinity ones. After this first, proximity-driven, accumulation of Xist, Xist then spreads to other target sites. This secondary spreading might be a consequence of the initial contacts, chromosome reorganization or intrinsic site affinity. Given that Xist spreading is likely to be a dynamic process, permissive sites might be those at which Xist is more stably retained [7, 13] (Fig. 1a). This model contrasts strongly with the classic model that proposes linear spreading of Xist along the X chromosome from the XIC [13, 14] (Fig. 1b).

Through nuclear architecture and topology studies of the X chromosome using the circular chromosome conformation capture technique (4C), Splinter and colleagues showed that, upon differentiation of female ESCs, the inactive X chromosome (Xi) loses the specific interactions between loci that are typical of the active X chromosome (Xa) [20]. This could be due to Xist binding sites differing in individual differentiated cells [7]. Differential Xist binding in individual cells might result in a loss of specific 4C signal at the level of the overall cell population upon Xist-induced chromatin remodeling. Noticeably, CHART and Xist-centered HiC profiles obtained at early time-points in differentiation do show some degree of overlap. However, while Xist profiles obtained by CHART and RAP-Seq are broad and diffuse [7, 8], the *Xist*-locus profile generated using 4C and HiC is quite sharp, suggesting only a few genomic locations are in close contact with the XIC [20]. This apparent discrepancy could reflect the preference of the latter technique for picking up those genomic sites that interact most frequently or are in closest proximity in most of the cells, while the CHART and RAP-Seq profiles more typically represent an ‘average’ signal of Xist contact sites within the overall population of cells [7, 8, 15, 16, 20]. Other differences might be reflections of different experimental protocols — Splinter and colleagues [20], for instance, used a differentiation protocol that enriches for a single specific cell lineage [neuronal precursor cells (NPCs)], whereas Simon et al. [8] and Engreitz et al. [7] used withdrawal of leukemia inhibitory factor (LIF) and differentiation by retinoic acid (RA), respectively. The latter two conditions are known to lead to the differentiation of a heterogeneous mixture of different cell types (Box 1).

Interestingly, Splinter et al. [20] and Minajigi et al. [21], provided evidence for the need for continuous Xist expression in order to achieve proper folding of the Xi. Indeed, a conditional deletion of *Xist* was shown to be associated with a reshaping of the topology of the Xi into an Xa-like conformation. Minajigi et al. also suggest a role for the cohesin complex in keeping Xa topologically associated domains (TADs) in place. Such conformation changes might explain the slightly higher rate of reactivation of X-linked genes in Xist-deficient cells observed by the Jaenisch group in the maintenance phase of XCI, which is otherwise thought to be Xist independent [22]. As Xist seems to interact directly with the lamin B receptor (LBR), a protein mediating the interaction between chromatin and lamin B [21, 23], this interaction could be a necessary intermediate in keeping the Xi in the proximity of the nuclear envelope, where heterochromatin is tethered, thereby reinforcing or stabilizing the Xi conformation and gene silencing [24] (Table 1).

**Table 1:** Factors involved in X chromosome inactivation

Factors involved in XCI	Function in the context of XCI	References
*Proteins*		
PRC2	The polycomb repressive complex 2 (PRC2) is known to be recruited early on the inactive X (Xi) during differentiation of embryonic stem cells (ESCs) and embryonic development and catalyzes methylation of histone H3 at K27 on chromatin	[40, 80, 81]
PRC1	The activity of polycomb repressive complex 1 (PRC1) on chromatin reinforces gene silencing by ubiquitylation of histone H2A at K119 and chromatin compaction. The order of recruitment of PRC2 and PRC1 to the Xi is still a matter of debate	[82, 83]
Saf-A (HnrnpU)	The Saf-A (HnrnpU) factor directly binds to Xist and mediates its interaction with chromatin through direct interaction with SARS/MARS elements	[21, 23, 44, 56, 58]
SHARP (Spen)	SHARP (Spen) directly binds to Xist and mediates the functional interaction between Xist and the NCoR complex	[21, 23, 44]
CTCF	The CCCTC-binding factor (CTCF) might work as a genomic insulator. In the context of X chromosome inactivation (XCI), it might serve as a barrier to Xist-induced chromatin reorganization	[21, 67]
SATB1	The special AT-rich sequence-binding protein-1 (SATB1) cellular regulator of higher chromatin organization has a role in the initiation of XCI. However, its precise role in XCI is not clear	[59, 84]
YY1	Yin-Yang 1 (YY1) is a bivalent protein with DNA-binding and RNA-binding motifs. It might have a role in tethering Xist to chromatin (spreading in cis) as well as a role in the regulation of Xist	[44, 60, 85]
SmchD1	The protein structural maintenance of chromosome hinge domain 1 (SmchD1) has a role in maintaining a correct pattern of DNA methylation on the Xi during the maintenance phase of XCI	[21, 86]
WTAP	Wilms’ tumor-associated protein (WTAP) is a splicing factor and interactor with Xist. It is involved in regulating RNA methylation. It might have a role in the post-transcriptional modification of Xist	[21, 23, 44]
LBR	The lamin B receptor (LBR) was recently identified as an Xist-binding protein. It is known to localize with the nuclear lamina and to interact with repressive complexes as well as with lamin B	[21, 23]
Rbm15	Rbm15 belongs to the SPEN family of transcriptional repressors and directly binds to Xist RNA	[23]
hnRNPK	Heterogeneous nuclear ribonucleoprotein K (hnRNPK) is an RNA-binding protein that interacts with Xist and plays a role in the Xist-mediated recruitment of repressive chromatin marks	[21, 44]
Oct4, Sox2, Rex1, Nanog, PRDM14, Klf4	Pluripotency factors and epigenetic regulators that have been shown to control XCI through the regulation of Xist and Tsix	[2, 74, 87, 88]
Rnf12	The Rnf12 protein seems to regulate the expression of Xist through degradation of Rex1	[75]
Atrx	The protein alpha thalassemia/mental retardation syndrome X-linked (Atrx) is involved in the recruitment of PRC2 on the inactive X chromosome	[21, 89]
*ncRNAs*		
Xist/Tsix	Xist is the master regulator of XCI, and Tsix is its major antagonist. Regulation of the levels of Xist and Tsix regulates the initiation of XCI	[2]
Jpx	The Jpx ncRNA seems to act as an activator of Xist	[2]
Ftx	The Ftx ncRNA seems to be an Xist activator	[2]
*Genomic elements*		
LINEs	The LINEs class of genomic repeats colocalize with inactive genes in the Xi territory and might have a role in the establishment and maintenance of XCI	[43, 90, 91]
SARS/MARS	Facultative scaffold/matrix attachment regions enriched in open chromatin and gene bodies where Xist accumulates	[7, 66]

An alternative method that has been applied to studying Xist localization and Xi topology is fluorescence microscopy. Smeets et al. [25] and Cerase et al. [26] have studied Xist localization by super-resolution three dimensional structured illumination microscopy (3D-SIM) [27], a technique that allows specimen imaging at sub-diffraction resolution (resolution limit ~100 nm; Box 1). Their findings challenge the idea of a broad distribution of Xist along the Xi and suggest that Xist, even when fully spread, might be in contact with only a limited number of genomic sites at any one time. Smeets and colleagues [25] have reported a discrete number of Xist foci (approximately 100 per cell) in fully differentiated and differentiating female ESCs. As each focus might represent multiple Xist molecules, the results are compatible with earlier estimates of the number of Xist molecules, which are in the range of 300 to 1000 per cell [28, 29]. The apparent disagreement with the results of chromosome-wide Xist profiling obtained by RAP and sequencing by capture hybridization analysis of RNA targets (CHART-seq) could reflect differences between the analysis of pooled and single cells. For example, it is clearly possible that Xist localizes to relatively few genomic locations at any one time in a given cell and yet appears as a broad domain when population-based sequencing approaches are used. An alternative explanation could be that single RNA molecules cannot be detected by RNA fluorescence in situ hybridization [25].

### Xist-mediated Polycomb recruitment and gene silencing

**Fig. 2:**
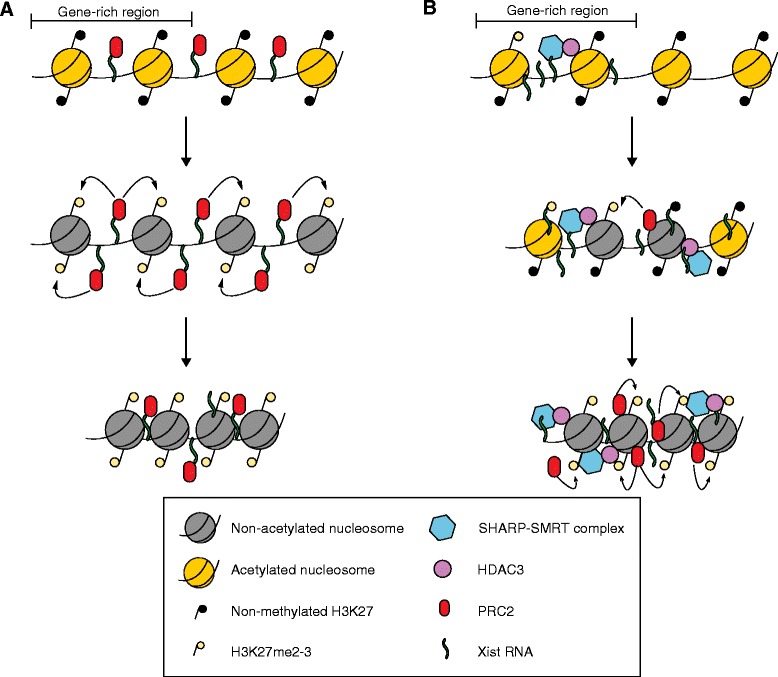
Direct and indirect models of recruitment of PRC2 by Xist RNA. **a** In the direct model, Xist localization brings PRC2 onto the chromatin by direct recruitment (*upper panel*). The PRC2 complex then places the H3K27me3 mark on the chromatin (*middle panel*), and this is followed by chromatin remodeler recruitment and chromatin compaction (*lower panel*). **b** In the indirect model, Xist interacts with gene-dense regions (*upper panel*) and induces chromatin changes (*middle panel*; i.e. histone deacetylation induced by Hdac3, chromatin compaction, eviction of RNA polymerase II). These changes might, in turn, recruit PRC1 or PRC2 and remodeler complexes (*lower panel*). *H3K27me2*-*3* dimethylated or trimethylated histone 3 lysine 27, *PRC1* polycomb repressive complex 1, *PRC2* polycomb repressive complex 2

A much-debated aspect of XCI is the link between Xist spreading and recruitment of Polycomb protein. The most widely accepted model predicts a direct recruitment of Polycomb by Xist RNA (Fig. 2a). This interaction has been reported to be mediated by the structurally conserved Xist RepA domain, which would interact directly with polycomb repressive complex 2 (PRC2) [30–33]. In agreement with this model, Engreitz et al. [7] and Simon et al. [8] found linear correlations between Xist and PRC2 localization and between Xist and PRC2-mediated tri-methylation of lysine 27 of histone H3 (H3K27me3). This agrees with previous mapping studies of PRC2 on the X chromosome that suggested a broad overall distribution of PRC2 and H3K27me3 [34–36] following accumulation at discrete sites (CpG islands) [36]. Most of the studies supporting a direct recruitment model [30–33] have, however, exploited in vitro biochemical approaches such as band-shift assays and RNA immunoprecipitation (RNA-IP) approaches (Box 1). Such techniques are notoriously prone to false-positive results, reflecting non-specific interactions between RNA and proteins.

An almost equally well-represented body of evidence argues against the direct-interaction model. For instance, some studies show that Xist upregulation clearly precedes PRC2 recruitment in early mouse development [37, 38], whereas others demonstrate that a RepA deletion mutant version of Xist (∆Arep) is still fully able to recruit PRC2 and H3K27me3 [37–40]. Still-other observations suggest that Xist expression in fully differentiated cells is not sufficient to recruit PRC2. The absence of PRC2 recruitment, upon Xist induction, is not related to the expression level of this complex, as the PRC2 complex is often expressed in such differentiated cells [41].

More-recent observations made by Cerase and colleagues [26] using a 3D-SIM approach showed that the bulk of Xist RNA and PRC2/PRC1 complexes are clearly spatially separated. This finding argues strongly against the direct-interaction model. The study used a mouse male ESC carrying an inducible *Xist* transgene (Xist-TG) inserted on chromosome 17 and capable of Xist upregulation under undifferentiated conditions. Whilst Xist spreading and localization could be influenced by this autosomal context [25, 26, 42, 43] and by the type of undifferentiated culture conditions used, the main findings of this paper have been confirmed using a fully differentiated female cell line [26].

The above study argues that the observed distance between Xist RNA and Polycomb proteins is likely to preclude direct interaction between the PRC complex and Xist (Fig. 2a). This interpretation is supported by results from Smeets et al. [25], who showed that Xist localizes to the inter-chromatin/perichromatin regions (IC/PR), a non-DAPI-dense area that displays poor overlap with H3K27me3 domains (correlating with DAPI-dense chromatin compartments) (Box 1). Both lines of evidence suggest an alternative model not only of how Xist might recruit Polycomb proteins, but also more generally of the role of chromatin remodelers in X inactivation (Fig. 2b). According to the model, in agreement with Engreitz et al. [7], Xist would initially interact with gene-dense silent regions, possibly partially marked by H3K27me3. It would then induce histone deacetylation, chromatin compaction and the exclusion of Pol II and the basal transcription machinery from nearby active regions [21, 23, 44]. Silenced, compacted chromatin would, in turn, recruit PRC2 and/or chromatin remodelers (indirect model; Fig. 2b) [23, 45, 46]. In accordance with a two-step recruitment model of chromatin factors by Xist, PRC2 initially would only accumulate at approximately 100 to 150 sites before spreading broadly along the X chromosome [36].

Interestingly, Simon and colleagues showed that, when Xist was stripped off the chromatin of female mouse embryonic fibroblasts (MEFs) using complementary locked nucleic acids (LNAs), the kinetics of re-attachment differed from that of the de novo kinetics [8]. This suggests that Xist could also function by priming the chromatin, possibly making it a better substrate for Xist re-spreading after cell division or for recruitment of repressive complexes (for example, by histone deacetylation) [8, 26, 39]. Such an interpretation is, however, in at least partial disagreement with a study from Ng and colleagues, where Xist re-spreading events were observed to occur with the same kinetics as those seen in the initial round of Xist spreading [47].

Very recently, indirect recruitment of PRC2 by Xist has been confirmed by two independent studies [23, 44], which used biotinylated probes complementary to Xist to pull down Xist-associated proteins for mass spectrometry analysis. While McHugh and colleagues used UV crosslinking conditions coupled with mass spectrometry under denaturing conditions (RAP-MS) [23], Chu and colleagues relied on formaldehyde crosslinking followed by mass spectrometry in non-denaturing conditions (ChIRP-MS) [44) (Box 1). The former technique allows recovery only of direct RNA–protein interactors, whereas the latter also allows recovery of proteins in the same complex or in close proximity that are not interacting directly with Xist [26]. The stringent conditions used by McHugh and colleagues allowed the specific isolation of ten bona fide Xist direct interactors. By contrast, Chu and colleagues found 81 proteins that directly or indirectly associate with Xist. Although Chu et al. reported a possible direct interaction with the PRC1 complex [44], neither study listed members of the PRC2 complex as Xist interactors. McHugh et al. suggest that PRC2 recruitment is a consequence of histone H3 deacetylation by Hdac3, part of the NCoR repressive complex, and exclusion of Pol II [23, 48]. They also suggest that the silencing mediator for the retinoic acid receptor and thyroid hormone receptor/nuclear receptor co-repressor (SMRT/NCoR) complex is recruited to the inactivating X via SMRT- and HDAC-associated repressor complex/Msx2-interacting protein (SHARP/Spen), which itself directly binds to Xist RNA [23, 44] (Fig. 2b). Both knockdown of Hdac3 and of SHARP/Spen have similar negative effects on PRC2 recruitment to the inactive X and gene silencing. Chu et al. also suggest that heterogeneous nuclear ribonucleoprotein K (HnrnpK), an heterogeneous nuclear ribonucleoprotein similar to Saf-A, but from which it differs in both binding sites and specificity, might have a direct role in Polycomb recruitment [44].

The results from another very recent proteomics paper [21] contrast with the findings of McHugh et al. and Chu et al. The authors, while using an approach similar to that used by McHugh et al. [22], describe 80 to 250 proteins interacting with Xist at any one time. Among these they were able to identify RbAp46/RbAp48 proteins as direct interactors with Xist. While these proteins are part of the repressive complex PRC2, it should be noted that they are also part of both the Nurd and Sin3 complexes [49].

Finally, roles have been proposed for Jarid2 and Pcl2, two non-canonical subunits of PRC2 [50], in mediating the recruitment of the PRC2 complex to the Xi [51, 52]. Knockdown and knockout experiments have shown that PRC2 recruitment on the X is impaired in the absence or reduction of these two PRC2 cofactors, whereas Xist upregulation itself seems to be unaffected. It should be noted that neither study [52, 53] allowed for discrimination between direct versus indirect PRC2 recruitment. Two interesting reviews have addressed the issue of Xist-mediated PRC2 recruitment in detail [54, 55].

### Nuclear scaffolding and XCI

Important examples of other putative Xist-interacting proteins that could be involved in Xist spreading and silencing include nuclear scaffold proteins [25, 56–60] (Table 1). The nuclear scaffold (also known as nuclear matrix) is a stable, proteinaceous structure that remains after treating cell nuclei with high-salt buffers, detergents and nucleases and might provide the framework for chromatin organization. In particular, scaffold-attachment or matrix-attachment regions (SARs or MARs) could be mediating the interaction between DNA and the matrix proteins in a highly regulated fashion.

**Fig. 3:**
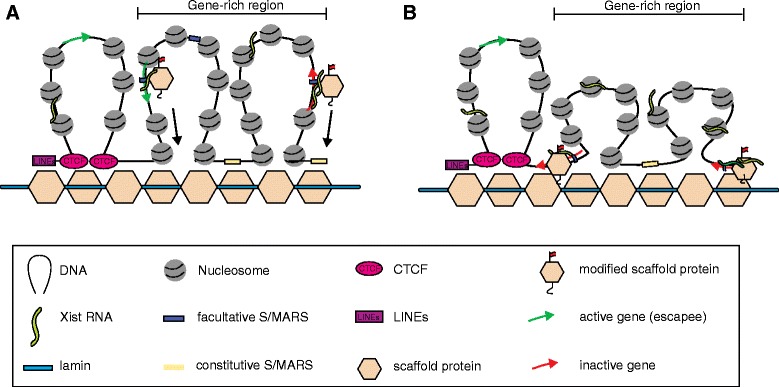
Possible role of scaffold proteins in X chromosome inactivation. **a** The binding of Xist to modified scaffold proteins induces the reorganization of the chromatin, as in (**b**), where Xist-mediated silencing is maintained by the nuclear scaffold. Genes to be silenced are dragged towards the nuclear matrix, preventing engagement of transcription factors at regulatory sites. CCCTC-binding factor (*CTCF*) might serve as a barrier to prevent Xist-induced chromatin reorganization. *LINEs* long interspersed nuclear elements

Fackelmayer and coworkers were the first to describe the enrichment of Saf-A, a nuclear matrix protein, in the Xi territory [56, 57]. Hasegawa and colleagues [58] subsequently showed that Saf-A is necessary for Xist localization in both neuroblasts and fully differentiated MEFs. They also showed, using UV crosslinking conditions and RNA-IP, that Xist and Saf-A might interact directly [58]. It should be noted that the UV crosslinking experiments performed by Hasegawa and colleagues are less prone to artifacts than are band-shift assays and non-crosslinked-formaldehyde IPs. This is because UV crosslinking between RNA and proteins is only effective over short distances [61] (Box 1). Nevertheless, nucleic-acid–protein and protein–protein interactions — involving not only Xist and Saf-A, but also other components — cannot be formally excluded. Smeet and colleagues [25], using a GFP–Saf-A fusion protein, have confirmed the enrichment of Saf-A on the Xi and have provided additional evidence of a direct interaction between Xist and the Saf-A protein. Using a 3D-SIM approach, they evaluated the average distance between Saf-A and Xist signals. Measured distances fall below the resolution limits of the technique, implying at least some degree of interaction. There is no reason to believe that the use of formaldehyde-fixed cells calls into question the observed interaction as Xist–matrix binding appears relatively stable [62, 63]. The results of Smeet and colleagues suggest that the Saf-A protein that is enriched on the Xi could be post-translationally modified (Fig. 3a), an observation in possible agreement with the lack of recognition of Saf-A on the Xi by certain antibodies against Saf-A [25]. The possibility of post-transcriptional modifications of the scaffold is of interest in the light of results concerning Xist spreading in cis and fuels speculation about the idea that Xist might interact with modified matrix proteins on the presumptive inactive X and that this would provide the mechanism for restricting Xist RNA spreading to the chromosome from which it was transcribed [64].

Chu et al. [44], McHugh et al. [23] and Minajigi et al. [21] all identified Saf-A as an interactor with Xist, using RAP-MS and ChIRP-MS, and confirmed the role of Saf-A in Xist localization on the inactive X, substantiating the previous findings of Hasegawa and colleagues [58]. Although the authors suggest a direct role for Saf-A in Xist-mediated silencing, as Xist silencing is affected both by a loss of Xist localization and by compaction, other possibilities must be considered [23, 44].

It is tempting to push this model slightly further and hypothesize that Xist interacts directly with modified scaffold proteins [63], mediating a chromosome-wide reorganization of the chromosome [40, 65]. Interestingly, facultative scaffold/matrix attachment regions (S/MARs) are enriched in open chromatin regions and in gene bodies, where Xist accumulates [7, 8, 66] (Box 1; Table 1). As Simons et al. and Engreitz et al. have shown that Xist does not accumulate on the gene body of escapee genes, which are genes that avoid being silenced by Xist, and active genes at early stages of XCI, we could further speculate that Xist needs to access the gene bodies to achieve full gene silencing. Under such a model, Xist would accumulate on S/MAR-enriched loci, interacting with post-translationally modified Saf-A, triggering the relocation of active genes close to repeat-dense regions (for example, LINE-rich and lamin-bound regions) [40, 66], in agreement with the model proposed by Chaumeil and colleagues [40] (Fig. 3a, b). Moving active genes into the proximity of the compacted/repeat-rich region of the Xi would prevent access of the transcription factors to regulatory regions of genes, resulting in silencing [35]. Escapee genes, through a looping of the chromatin outside and away from the repeat-dense region, would be protected from silencing [40, 67]. CCCTC-binding factor (CTCF) is one factor that has been reported to have a role in the organization of chromosomal domains that efficiently escape XCI [67] (Table 1). The Smeets et al. paper [25], however, challenges the common view of a compacted central area of the Xi, with active genes arranged at the periphery of the Xi [40, 63]. Instead, Smeets and colleagues suggest a honeycombed structure, with the center of the Xi marked by pockets of reduced compaction, which are permissive for transcription [25]. Differences in the proposed structures likely reflect differences in microscope resolution.

**Fig. 4:**
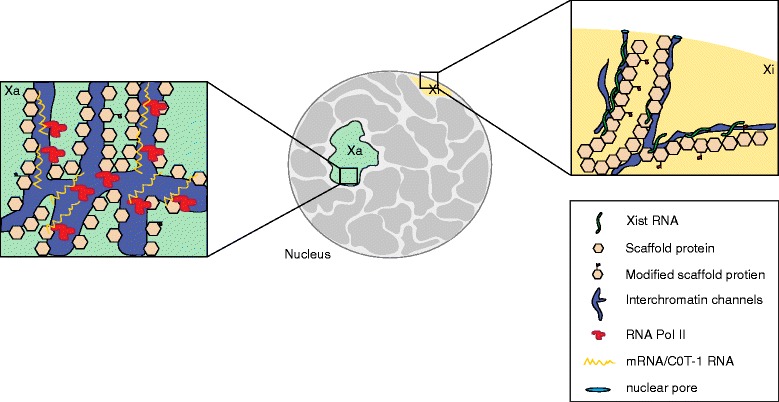
A speculative model of Xist function. The central part of the diagram shows a nucleus, with the active (Xa) and the inactive (Xi) chromosomal territories highlighted in *green* and *yellow*, respectively (*gray* indicates the chromosomal territories of other chromosomes). Magnified views of the Xi (*right*) and the Xa (*left*) territories are shown. The following model is based on the observations of Smeets and colleagues [25]. Coating with Xist RNA might cause a collapse of open chromatin channels, and this, in turn, might block the access of transcription factors and RNA polymerase II (*RNA Pol II*) to gene-regulatory elements. Alternatively, Xist might compete with C0t-1 RNA, and removal of this class of RNA could, in turn, lead to chromosome compaction [68]

A very recent paper [68] also hints at a possible role of the nuclear matrix in more-general chromatin organization, suggesting that the interaction of non-coding RNAs (ncRNAs) and the nuclear matrix might be a more widespread phenomenon. The authors showed an enrichment of C0T-1 RNA in euchromatic regions that directly interact with the nuclear matrix. Such interspersed repeat RNA, which mostly consists of truncated 5′ L1 elements, could serve either to recruit transcription factors or to act as a platform for opening up the chromatin. We are tempted to speculate that Xist competes with C0t-1 RNA for scaffold attachment sites in this context, triggering a release of structural euchromatic RNA, a collapse of chromatin, and triggering silencing [68] (Fig. 4a, b). Smeets and colleagues offer a similar potential explanation for the repressive function of Xist, linked to Xist coating of the Xi, which would prompt a collapse of interchromatin channels that, in turn, impedes access of Pol II and basal transcription factors to the chromatin. The absence of transcription would, in turn, trigger the recruitment of repressive complexes (for example, PRC2/PRC1 and DNA methyltransferases), inducing further silencing (Fig. 4a, b). For more information about the role of scaffolding in XCI, we refer the reader to two recent reviews [69, 70].

## Concluding remarks

Here, we have discussed several notable advances in the field of Xist biology. The articles under review represent important contributions to our understanding of the mechanism(s) of Xist silencing, especially in relation to four main areas for which there are outstanding gaps in knowledge: (1) Xist spreading; (2) Xi nuclear organization; (3) Polycomb/chromatin remodeler recruitment and gene silencing; and (4) Xist–matrix interactions.

The very recent papers of McHugh et al. [23], Chu et al. [44] and Minajigi et al. [21] have critically shed light on the previously elusive Xist-interacting proteins and how Xist might both establish gene silencing by Hdac3-mediated histone deacetylation and reinforce gene silencing by tethering the inactive X chromosome to the nuclear periphery through interaction with the lamin B receptor (LBR) and topoisomerase remodeling of the Xi.

However, many unresolved questions remain. For example, currently available data do not allow determination of whether different Xist splicing variants have a similar function and spreading pattern compared with those of the full-length Xist RNA that is most often exploited experimentally.

Several Xist splice variants have been described, including two major forms [71–73]. More recently a RepA variant of Xist RNA that seems to mimic the full-length version has also been reported [30]. Use of the male ESC Xist-inducible system, which exploits a mature form of Xist, and differentiating female ESC lines, in which Xist transcription is subject to splicing, might therefore not necessarily be completely interchangeable experimentally. A possible way to make the systems more comparable would be to include the relatively small introns of Xist in the inducible constructs. This could be particularly informative in the light of eventual Xist post-transcriptional modifications, which are not necessarily confined to exons.

The regulation of Xist has been shown to be tightly regulated by pluripotency factors [6, 74, 75], and the pluripotent state of ESCs is known to be highly sensitive to culture conditions. If, as seems likely, silencing initiation and Xist spreading are sensitive to quantitative variations in Xist RNA levels, ex vivo culture conditions might also crucially impact inactivation parameters. For example, ESCs grown in 2 inhibitor (2i) medium culture conditions are known to be closer to the ‘ground zero’ state of pluripotency [76], to have a more homogeneous composition [76] and to show different transcriptional profiles compared with those of cells grown under conventional LIF and serum conditions [77–79]. Both differentiation and upregulation of XIC lncRNAs clearly occur much faster using such 2i cultured cells, but whether the underlying mechanisms differ or remain unchanged still needs to be clarified. Only through standardization of ESC culturing (for example, by consistent use of 2i culturing conditions) and differentiation protocols (for example, using NPC differentiation) will direct comparison of data obtained in different experiments be possible.

Finally, given that the initial Xist spreading is likely to vary between individual cells, validation at the single-cell level, including single-cell RAP/CHART experiments, will be key towards obtaining a more thorough characterization and a better knowledge of Xist early dynamics. Such approaches would be expected to facilitate the identification of causal correlations between possible chromatin states and specific modifications of Xist binding sites.

## Abbreviations

2C: two-cell embryonic stage
2i medium: 2 inhibitor medium
3D-SIM: three dimensional structured illumination microscopy
4C: circular chromosome conformation capture
Atrx: alpha thalassemia/mental retardation syndrome X-linked
CHART: capture hybridization analysis of RNA targets
ChIRP-MS: chromatin isolation by RNA purification-mass spectrometry
CLIP: crosslinking and immunoprecipitation
CTCF: CCCTC-binding factor
DAPI: 4′,6-diamidino-2-phenylindole
EB: embryoid body
ESC: embryonic stem cell
Hi-C: high-throughput chromosome conformation capture
HnrnpK: heterogeneous nuclear ribonucleoprotein K
H3K27me3: histone 3 lysine 27 trimethylation
IP: immunoprecipitation
lncRNA: long non-coding RNA
LBR: lamin B receptor
LIF: leukemia inhibitory factor
LINE: long interspersed nuclear element
MEF: mouse embryonic fibroblast
ncRNA: non-coding RNA
NPC: neuronal precursor cell
Pol II: RNA polymerase II
RA: retinoic acid
RAP-Seq: RNA antisense purification-sequencing
RAP-MS: RNA antisense purification-mass spectrometry
RepA: repeat A region of Xist RNA
RNA-IP: RNA immunoprecipitation
Pol II: RNA polymerase II
PRC1/PRC2: polycomb repressive complex 1/polycomb repressive complex 2
SAF-A (hnrnpU): scaffold attachment factor A (heterogeneous ribonucleoprotein U)
SatB1: special AT-rich sequence-binding protein-1
SINE: short interspersed nuclear element
SmchD1: structural maintenance of chromosome hinge domain 1
SHARP/Spen: SMRT- and HDAC-associated repressor complex/Msx2-interacting protein
S/MAR: scaffold/matrix attachment region
SMRT/NCoR: silencing mediator for retinoic acid receptor and thyroid hormone receptor/nuclear receptor co-repressor
TAD: topologically associated domain
WTAP: Wilms’ tumor-associated protein
Xa: active X chromosome
XCI: X chromosome inactivation
Xi: inactive X chromosome
XIC: X inactivation center
Xist: inactive X-specific transcript
Xist-TG: Xist transgene
YY1: Yin-Yang 1
